# Immediate-use steam sterilization sterilizes N95 masks without mask damage

**DOI:** 10.1017/ice.2020.145

**Published:** 2020-04-17

**Authors:** Irene O. Carrillo, Anna C.E. Floyd, Christian M. Valverde, Travis N. Tingle, Firas R. Zabaneh

**Affiliations:** 1 Houston Methodist Research Institute, Houston, Texas; 2 Houston Methodist Hospital, Houston, Texas

*To the Editor—*As coronavirus disease 2019 (COVID-19) cases increase around the world, severe shortages of essential healthcare supplies are hampering the efforts to provide life-saving medical care without compromising the safety of healthcare workers. COVID-19 is transmitted by droplets, meaning that clinicians can be protected by gloves, gowns, eye shields, and masks. Disposable N95 masks seal tightly to the face and provide significantly better protection than surgical masks.

Established infectious disease protocol requires that clinicians dispose of N95 masks after contact with a patient. However, the United States will need 3.5 billion N95 masks for healthcare workers during this pandemic, and currently has ~1% of that necessary volume.^1^ The Center for Disease Control and Prevention recommends that clinicians save masks and reuse them, and clinicians are currently using N95 masks for full shifts.^2^ With each attempt to safely don a contaminated N95 mask, the risk for infection of vital clinicians grows. In countries where equipment shortages have progressed, healthcare workers are currently being infected with SARS-CoV-2 at 3 times the rate of the general population, which reduces the ability of hospitals to provide adequate care and increases COVID-19 patient death rates. Thus, it is essential to create a protocol for sanitizing masks without reducing efficacy.

N95 masks are composed of thermoplastic elastomer (straps), aluminum (nose clip), polyurethane (nose foam), polypropylene (filter), and polyester (shell and cover web). The polyester shell and cover web are created from disorganized, thin fibers with an electrostatic charge. This design, while effective for reducing infection in clinicians, poses significant challenges for sanitization. Washing these masks with water decreased performance by 21%.^3^ Sanitizing N95 masks with alcohol similarly reduced performance by 37% and resulted in significant shrinkage.^4^ Ultraviolet germicidal irradiation has been tested for sanitization of N95 masks, but in 90% of cases, the integrity of the masks was compromised.^5,6^ Sanitization by bleach or ethylene oxide created significant risk to mask wearers due to residue left on the mask.^6^ Thus, novel methods of sanitizing N95 masks to ensure safety of clinicians working in factious disease units is needed.

Immediate-use steam sterilization (IUSS), using a Steris Amsco Evolution HC1500 PreVac Steam Sterilizer autoclave (Steris, Mentor, OH) was performed on N95 masks. Masks were packed in paper-plastic sterilization peel pouches for IUSS (Medical Action Industries 8” role, no. 422R). Masks were photographed and fit tested prior to IUSS, and this testing protocol was repeated after the IUSS cycle. The 3M 1870 and M3 1870+ masks (3M, Saint Paul, MN) retained efficacy in a quantitative fit test. Quantitative fit tests were performed using the gold standard TSI PortaCount Respirator Fit Tester (TSI, Shoreview, MN).

**Fig. 1.**
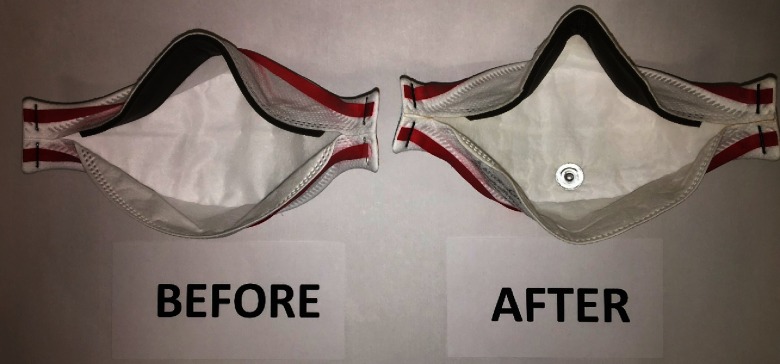
N95 masks retained structural integrity after 3 immediate-use steam sterilization (IUSS) cycles.

We tested 5 subjects to investigate individual differences between faces. For each subject, a fit test was performed before the IUSS cycle as a control. Fit tests were performed again after 3 IUSS cycles. In all cases, masks retained their structural integrity and efficacy (Fig. 1).

The IUSS cycle is performed with a chemical indicator (3M Comply SteriGage Steam Chemical Integrator, no. 422R) and a biological indicator (3M Attest Super Rapid 5 Steam Plus Callenge Pack, no. 41482V) for every autoclave cycle, confirming that no biological or chemical contamination is present on the masks. If either indicator fails the IUSS cycle, the masks are reprocessed and are not placed into the hospital system.

Our stuy was limited by a small sample size. Follow-up studies will be conducted with a significantly larger sample by recruiting participants from the Houston Methodist Hospital who will be fit tested for their mask each day.

Despite the limitations of this study, the data herein provide a valid basis for the use of IUSS for N95 masks to prevent the spread of SARS-CoV-2 to healthcare workers.
